# Clinical effects, cardiovascular and renal outcomes associated with rapid-acting insulin analogs among individuals with type 2 diabetes: a nation-wide observational cohort study

**DOI:** 10.1186/s40842-017-0043-2

**Published:** 2017-06-19

**Authors:** Ann-Marie Svensson, Mervete Miftaraj, Stefan Franzén, Björn Eliasson

**Affiliations:** 1 Center of Registers in Region Västra Götaland, Gothenburg, Sweden; 2 Department of Medicine, University of Gothenburg, Gothenburg, Sweden; 3 Department of Medicine, Sahlgrenska University Hospital, S-423 45 Gothenburg, Sweden

**Keywords:** Type 2 diabetes mellitus, Insulin analog, Mortality, Cardiovascular disease

## Abstract

**Background:**

Rapid-acting insulin analogs (RAIs) have not been examined for long-term safety in randomized clinical trials. We performed a nationwide longitudinal cohort study among individuals with type 2 diabetes (T2DM) to address cardiovascular safety and mortality among users of lispro, aspart and glulisine insulins.

**Methods:**

We used four national registers, following patients previously not treated with RAI but with continuous use of RAIs in 2005-2014 up to 6.4 years, to examine HbA1c and weight, and the occurrence of severe hyperglycemia or hypoglycemia, renal failure, cardiovascular events or death. The treatment groups were compared using a weighted Cox proportional hazards model.

**Results:**

We included 17,620 patients, mean age slightly higher than 60 years, diabetes duration 9.9–11.7 years, mean BMI 30.5 kg/m^2^, HbA1c around 70 mmol/mol (8.6% NGSP), and 40.9–54.0% of the patients exhibiting eGFR <60 ml/min/1.73 m^2^ in the three groups. Around 95% of the patients also used another insulin, and 24.2–24.7% had a history of cardiovascular disease (CVD).

Mean HbA1c and weight levels were stable and similar. Incidence rates of death were 234.4, 284.9 and 156.7 per 1000 person-years among users of lispro, aspart, and glulisine; incidence rates of all cardiovascular events were 668.4, 622.4, and 699.5 per 1000 person-years, respectively.

There were no differences in mortality, CVD, renal failure or severe hypoglycemia or hyperglycemia, although a lower mortality risk in patients on glulisine compared with aspart, and lower risk of stroke in users of glulisine was suggested. The risk of severe hyperglycemia was higher with lispro than aspart, and lower of severe hypoglycemia than aspart or glulisine among the older age group.

**Conclusions:**

Overall, there do not appear to be any major important differences in effects on hypoglycemia, hyperglycemia, weight or long-term safety between the three available RAIs among insulin-naive individuals with T2DM in clinical practice.

## Background

Several pharmacological treatments may be considered for type 2 diabetes, but some patients eventually require and benefit from insulin treatment [1]. Basal insulins are often used in combination with oral hypoglycemic agents or GLP-1 receptor analogs, but rapid-acting insulin analogs (RAIs) may be required to prevent postprandial glucose excursions. Non-inferiority and some clinical advantages over ordinary human insulin (fewer doses, increased flexibility, less hypoglycemia and improved cost-effectiveness) have been documented [2], but only a few studies have directly compared the clinical efficacy of various RAIs [3–6], and none have addressed long-term cardiovascular safety.

We recently performed a population-based longitudinal cohort study among individuals with type 1 diabetes to address cardiovascular safety and mortality among continuous users of the currently available RAIs (lispro, aspart and glulisine insulins) [7]. There were no pronounced differences in effectiveness or long-term safety between the three options, although severe hypoglycemia was more common among older patients, while severe hypoglycemia and hyperglycemia were more common among patients with impaired renal function. In a manner that similarly linked data from the Swedish National Diabetes Register (NDR) with other databases to capture information about hospitalization and cause of death, the present study examined long-term, real-life safety data concerning the impact of RAIs among individuals with type 2 diabetes, including elderly patients and those with renal impairment.

## Methods

The regional ethical review board approved this study, which was carried out in accordance with the Declaration of Helsinki. All patients had given informed consent to participation.

### Databases

We used data from several national sources. NDR is a quality registry with nationwide coverage [8], to which clinical characteristics, treatments and complications are reported annually by hospitals and primary healthcare centers. The Prescribed Drug Register (PDR) covers all prescriptions filled at pharmacies, while the Cause of Death Register contains data about mortality and date of death, while the National Patient Register focuses on hospitalization, procedures and diagnoses (Swedish National Board of Health and Welfare). The Longitudinal Integration Database for Health Insurance and Labor Market Studies is a source for place of birth and educational level. These databases have recently been reviewed and validated [9].

### Patients, study period, follow-up and censoring

**Fig. 1:**
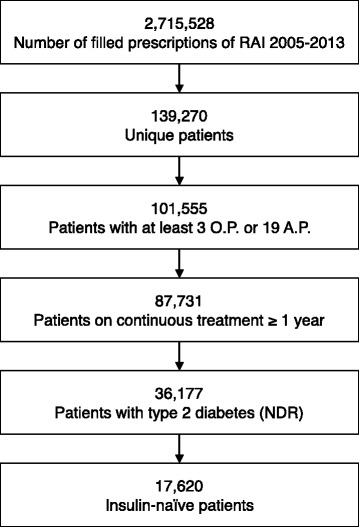
Patient selection flowchart. Data from the national pharmacy register. NDR, National Diabetes Register. O.P. ordinary prescription, A.P. ApoDos prescription

We identified all persons who had filled prescriptions of RAI, and thereafter included in this study patients with type 2 diabetes (clinical diagnosis as determined by the reporting physician) who were at least 18 years old. The study period was from July 1, 2005 (start date of PDR) to December 31, 2014. The first RAI prescription filled in the PDR after July 1 2005 was defined as the index date. Only patients not previously treated with RAIs (from July 1, 2005 to the index date) but who had used RAIs continuously for at least 1 year after the index date, were included in the study (Fig. 1). Previously filled prescriptions of a basal insulin were allowed. We defined continuous use as having filled at least three ordinary or 19 multidose (ApoDos) prescriptions during the subsequent 12 months. Patients were monitored the entire study period or until the occurrence of a censoring event. The start of the follow-up period was defined as the date that the third insulin prescription was filled. We used the following censoring events: picking up a new type of RAI, death, emigration to another country or the occurrence of a safety outcome.

### Patient characteristics

Baseline (index date) variables included age, gender, duration of diabetes, smoking, physical inactivity (exercising less than once a week), higher (postsecondary) education, history of hyperglycemia or hypoglycemia, history of cardiovascular disease (CVD), glucose-, lipid- and blood pressure-lowering medications, platelet aggregation inhibitors and anticoagulants, as well as type of basal insulin. We used the latest values for HbA1c, BMI, weight, blood pressure, blood lipids (total cholesterol, LDL cholesterol, HDL cholesterol and triglycerides), and estimated glomerular filtration rate (eGFR) up to 12 months before the index date. The eGFR was calculated using the Modification of Diet in Renal Disease study equation [10], and renal impairment was defined as eGFR <60 mL/min/1.73m^2^. HbA1c analyses and other laboratory tests were performed locally.

We defined a history of coronary heart disease (CHD) as diagnosis of ischemic heart disease (I20–I25) or treatment with percutaneous coronary intervention or coronary artery bypass graft prior to the index date (Table 1). Similarly, history of cardiovascular disease (CVD) was defined as diagnosis of stroke or peripheral vascular disease prior to the index date or history of CHD. We used ICD-codes to determine a history of atrial fibrillation, congestive heart failure, stroke, kidney failure, severe hyperglycemia and hypoglycemia.

**Table 1:** ICD groups and corresponding codes

Group number and name	ICD diagnosis or treatment codes
0. Ischemic heart disease	I20–25
1. PCI	FNG0, FNG00, FNG02, FNG05, FNG06, FNG10, FNG30, FNG96
2. CABG	FNA0, FNA00, FNA10, FNA20, FNA96, FNB00, FNB20, FNB96, FNC10, FNC20, FNC30, FNC40, FNC50, FNC60, FNC96, FND10, FND20, FND96, FNE00, FNE10, FNE20, FNE96, FNF00, FNF10, FNF20, FNF30, FNF96
3. Stroke	I61, I63, I64, I67.9
4. Peripheral vascular disease	I70.2, I73.1, I73.9, I79.2, E10.5, E11.5, E14.5. NHQ09, NHQ11, NGQ09, NGQ11, NGQ99, NFQ09, NFQ19, NFQ99, NEQ19, NEQ99
5. Atrial fibrillation	I46–48
6. Congestive heart failure	I50
7. Kidney failure	N18, N19
8. Hypoglycaemia	E100, E106A, E110, E110C, E110X, E116A, E120, E130, E140, E159, E160, E161W, E162, R402
9. Hyperglycaemia	E100A, E100B, E100D, E100X, E101, E101A, E101B, E101D, E101X, E110, E110A, E110B, E110D, E110X, E111, E111A, E111B, E111D, E111X, R739

A. CHD = groups 0–2

B. CVD = groups 0–4

### Outcomes

We followed the annual changes in HbA1c and weight, as well as the occurrence of safety outcomes (hospitalization due to hyperglycemia or hypoglycemia, renal failure, cardiovascular events – CHD, CVD, stroke, atrial fibrillation or congestive heart failure – or death), according to Table 1.

### Statistical methods

Missing baseline data were imputed using multiple changed equations, creating ten data sets [11]. The percentage of missing data ranged from zero (age, gender, treatment and prior conditions) to 76% (physical activity).

We used a multinomial generalized boosted regression model [12] with a logistic link function to estimate propensity scores [13] based on all baseline characteristics and risk factor treatments for each imputed data set. The inverse probability of the given treatment was used to weight differences in baseline characteristics between treatment groups.

Patient characteristics at baseline were summarized by means of standard descriptive statistics. Variations between the treatment groups were evaluated graphically based on the maximal pairwise standardized difference. Unadjusted mean HbA1c and weights were plotted by means of penalized B-splines with 95% confidence intervals (CI). The treatment groups were compared with respect to cardiovascular events and mortality using a weighted Cox proportional hazards model with robust standard errors that reflected the weighted analysis [14]. We used SAS version 9.4 and R version 3.1.0 to perform the statistical calculations.

## Results

We followed 17,620 patients with type 2 diabetes who used RAIs continuously for up to 6.4 years. There were numerical differences in baseline characteristics between the treatment groups before inverse probability of treatment weighting (Table 2). Prior to weighting only age and duration differed between treatments with a maximal pairwise standardized difference (SD) of 10.2 and 11.5%, respectively. After the weights were applied, the SD decreased to less than 2.4%, demonstrating perfect balance between the three treatment groups. Overall, around 40% were female and the mean age was slightly higher than 60, 41.9–49.2% of the patients being age 65 or older, and diabetes duration between 9.9 and 11.7 years. The mean BMI was 30.5 kg/m^2^, HbA1c around 70 mmol/mol (8.6% National Glycohemoglobin Standardization Program), total cholesterol 4.7 mmol/l, serum triglycerides 2.2 mmol/l and HDL cholesterol 1.2 mmol/l, while mean blood pressure was around 135/76 mmHg. Mean serum creatinine was around 84, eGFR 82 ml/min/1.73 m^2^, with between 40.9% and 54.0% of the patients exhibiting eGFR <60 ml/min/1.73 m^2^. There were small differences in the percentage of smokers, physical inactivity and education level, as well as in use of oral hypoglycemic agents, GLP-1 receptor agonists and lipid-lowering medication. Around 95% of the patients also used another type of insulin, but only 12 patients used insulin pumps (five lispro, six aspart, one glulisine). A history of cardiovascular disease was common and similar in the three treatment groups (24.2–24.7%), as was the case with atrial fibrillation and congestive heart failure (CHF), while the proportion of patients who had been hospitalized due to severe hypoglycemia or hyperglycemia was only 2.0–3.6% (Table 2).

**Table 2:** Baseline characteristics of patients treated with lispro, aspart and glulisine

	Lispro (*n* = 1986)	Aspart (*n* = 14,501)	Glulisine (*n* = 1133)
Age, years (SD)	60.8 (12.9)	60.9 (12.4)	63.2 (10.8)
Age 65 years or older, n (%)	1861 (43.4%)	6082 (41.9%)	558 (49.2%)
Female, n (%)	796 (40.1)	5460 (37.7)	445 (39.3)
Diabetes duration, years (SD)	10.8 (8.2)	9.9 (8.1)	11.7 (7.6)
Total cholesterol, mmol/L (SD)	4.7 (1.1)	4.7 (1.1)	4.6 (1.1)
Triglycerides, mmol/L (SD)	2.2 (1.5)	2.2 (1.5)	2.2 (1.7)
HDL, mmol/L (SD)	1.2 (0.4)	1.2 (0.4)	1.2 (0.4)
HbA1c, mmol/mol (SD)	70.9 (17.0)	69.6 (16.6)	70.4 (16.6)
BMI, kg/m^2^ (SD)	30.5 (4.9)	30.5 (4.9)	30.6 (5.1)
Systolic BP, mmHg (SD)	134.6 (15.7)	136.0 (16.7)	135.8 (15.7)
Diastolic BP, mmHg (SD)	76.7 (9.7)	76.9 (9.8)	76.1 (9.9)
Serum creatinine, x (SD)	82.7 (33.8)	84.6 (38.4)	83.9 (32.7)
eGFR, mL/min/1.73m^2^ (SD)	83.5 (36.2)	82.3 (29.1)	81.5 (27.7)
Renal impairment, n (%)	911 (45.9%)	5924 (40.9%)	612 (54.0%)
Smokers, n (%)	164 (14.1%)	1176 (15.5%)	101 (12.8%)
Higher education, n (%)	405 (20.7%)	2531 (17.7%)	206 (18.5%)
Physical inactivity, n (%)	307 (30.8%)	2052 (31.7%)	196 (28.7%)
Oral hypoglycemic agent, n (%)			
Metformin	1433 (72.2%)	9805 (67.6%)	865 (76.3%)
Sulphonylurea/meglitinide	672 (33.8%)	5316 (36.7%)	514 (45.4))
Thiazolidindione	97 (4.9%)	822 (5.7%)	87 (7.7%)
DPP-4 inhibitor	149 (7.5%)	656 (4.5%)	102 (9.0%)
Acarbose	27 (1.4%)	201 (1.4%)	22 (1.9%)
GLP-1 receptor agonist	104 (5.2%)	324 (2.2%)	63 (5.6%)
Insulin pump (CSII), n (%)	5 (0.6%)	6 (0.1%)	1 (0.2%)
Other insulin, n (%)	1865 (93.9%)	13,780 (95.0%)	1090 (96.2%)
Lipid-lowering agent, n (%)	1282 (64.6%)	9149 (63.1%)	841 (74,2%)
History of …, n (%)			
Ischemic heart disease	204 (12.1%)	1561 (10.8%)	123 (10.9%)
Atrial fibrillation	167 (8.4%)	1306 (9.0%)	82 (7.2%)
Myocardial infarction	182 (9.2%)	1344 (9.3%)	108 (9.5%)
Unstable angina	298 (15.0%)	2032 (14.0%)	163 (14.4%)
PCI	176 (8.9%)	1145 (7.9%)	83 (7.3%)
CABG	95 (4.8%)	750 (5.2%)	52 (4.6%)
Peripheral vascular disease	41 (2.1%)	469 (3.2%)	27 (2.4%)
Stroke	106 (5.3%)	693 (4.8%)	44 (3.9%)
Congestive heart failure	158 (8.0%)	1187 (8.2%)	71 (6.3%)
Hypoglycaemia	39 (2.0%)	306 (2.1%)	24 (2.1%)
Hyperglycaemia	64 (3.2%)	516 (3.6%)	30 (2.6%)

Means, standard deviations (SD), proportions; BP, blood pressure

**Fig. 2:**
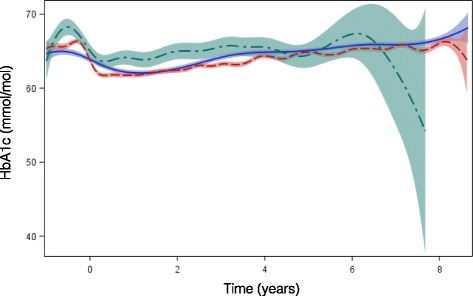
Average HbA1c during follow-up period among patients treated with lispro, aspart, and glulisine

**Fig. 3:**
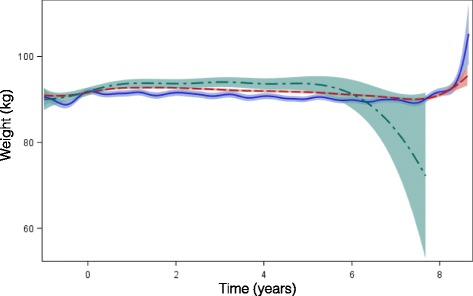
Average weight during follow-up period among patients treated with lispro, aspart, and glulisine

The mean follow-up periods were 2.0, 2.9 and 1.9 years among patients treated with insulins lispro, aspart and glulisine, respectively. Unadjusted mean HbA1c and weight during the follow-up period were stable and very similar (Figs. 2 and 3). The absolute numbers and incidence rates of death, cardiovascular events, CHF, renal failure and severe hypoglycemia or hyperglycemia are shown in Table 3. The incidence rates of death were 234.4, 284.9 and 156.7 per 1000 person-years among users of lispro, aspart, and glulisine, respectively, while the incidence rates of all cardiovascular events were 668.4, 622.4, and 699.5 per 1000 person-years, respectively.

**Table 3:** Follow-up period, number of events and incidence per 1000 person-years

Event	Lispro(*n* = 1986)	Aspart(*n* = 14,501)	Glulisine(*n* = 1133)
Deaths	93 (234.4)	1192 (284.9)	33 (156.7)
Fatal CHD	29 (73.1)	352 (84.1)	13 (61.7)
Fatal CVD	34 (85.7)	424 (101.3)	14 (66.5)
CHD	196 (546.9)	1896 (489.9)	110 (571.9)
CVD	234 (668.4)	2312 (622.4)	132 (699.5)
Stroke	39 (99.7)	412 (100.1)	11 (52.6)
Heart failure	109 (289.0)	1177 (295.5)	46 (225.7)
Kidney failure	75 (193.8)	708 (173.4)	28 (135.8)
Hypoglycemia	15 (38.1)	234 (56.5)	12 (57.6)
Hyperglycemia	10 (25.3)	190 (45.8)	7 (33.4)
Maximum follow up time, years	6.3	6.4	6.1
Mean follow up time, years	2.0	2.9	1.9
Median follow up time, years	1.3	2.9	1.8

Number of events (incidence per 1000 person-years). CHD coronary heart disease, CVD cardiovascular disease

We used Cox regression analyses to distinguish differences among the treatment groups with respect to mortality, CVD, CHF, renal failure and risk of hypoglycemia or hyperglycemia that required hospitalization (Tables 4, 5 and 6). There were no statistically significant differences, apart from a lower relative mortality risk among patients on insulin glulisine than on insulin aspart, lower risks of stroke among users of glulisine, and higher risk of severe hyperglycemia among aspart than lispro users. For patients age 65 or older, these results were virtually unchanged, but users of lispro were also at lower risk of severe hypoglycemia. Among patients with renal impairment (Table 6), there were no significant differences between the treatment groups.

**Table 4:** Risks of death and hospitalisations

Event	Aspart vs lispro	Glulisine vs lispro	Glulisine vs aspart
Total Mortality	1.23 [0.98, 1.53]	0.65 [0.41, 1.53]	0.53 [0.35, 0.8]*
CHD	0.96 [0.82, 1.12]	1.03 [0.79, 1.36]	1.08 [0.85, 1.35]
Fatal CHD	1.17 [0.79, 1.74]	1.04 [0.50, 2.16]	0.89 [0.47, 1.67]
CVD	0.95 [0.82, 1.09]	1.03 [0.80, 1.31]	1.08 [0.88, 1.33]
Fatal CVD	1.15 [0.80, 1.66]	0.88 [0.44, 1.78]	0.77 [0.41, 1.42]
Stroke	0.94 [0.67, 1.34]	0.33 [0.16, 0.66]*	0.34 [0.18, 0.65]*
Heart Failure	1.00 [0.82, 1.23]	0.76 [0.50, 1.13]	0.75 [0.53, 1.08]
Kidney Failure	0.88 [0.68, 1.13]	0.71 [0.42, 1.20]	0.81 [0.50, 1.30]
Hypoglycaemia	1.42 [0.83, 2.44]	1.30 [0.55, 3.10]	0.92 [0.45, 1.85]
Hyperglycaemia	1.97 [1.01, 3.85]*	1.88 [0.66, 5.33]	0.96 [0.42, 2.18]

Weighted Cox proportional hazards analysis. Estimated hazard ratios with 95% confidence intervals. * *p*-value <0.05

**Table 5:** Risks of death and hospitalisations: subgroup analysis in patients 65 years or older

Event	Aspart vs lispro	Glulisine vs lispro	Glulisine vs aspart
Total Mortality	1.10 [0.84, 1.44]	0.61 [0.37, 1.02]	0.56 [0.36, 0.87]
CHD	1.02 [0.83, 1.26]	1.09 [0.78, 1.52]	1.06 [0.81, 1.40]
Fatal CHD	1.18 [0.74, 1.89]	0.85 [0.38, 1.90]	0.72 [0.37, 1.40]
CVD	0.95 [0.78, 1.14]	0.98 [0.72, 1.33]	1.04 [0.80, 1.34]
Fatal CVD	1.15 [0.75, 1.78]	0.76 [0.36, 1.61]	0.66 [0.35, 1.23]
Stroke	0.86 [0.56, 1.33]	0.28 [0.11, 0.71]*	0.32 [0.14, 0.76]*
Heart Failure	0.97 [0.75, 1.25]	0.74 [0.47, 1.18]	0.77 [0.51, 1.15]
Kidney Failure	0.78 [0.58, 1.06]	0.68 [0.38, 1.22]	0.87 [0.52, 1.46]
Hypoglycaemia	2.37 [1.07, 5.25]*	3.24 [1.12, 9.42]*	1.37 [0.65, 2.88]
Hyperglycaemia	4.67 [1.49, 14.65]*	0.39 [0.09, 1.64]	0.39 [0.09, 1.64]

Weighted Cox proportional hazards analysis. Estimated hazard ratios with 95% confidence intervals. * *p*-value <0.05

**Table 6:** Risks of death and hospitalisations: subgroup analysis in patients with renal impairment

Event	Aspart vs lispro	Glulisine vs lispro	Glulisine vs aspart
Total Mortality	0.97 [0.61, 1.54]	0.56 [0.21, 1.50]	0.58 [0.24, 1.40]
CHD	1.05 [0.70, 1.60]	0.96 [0.51, 1.81]	0.91 [0.55, 1.50]
Fatal CHD	1.17 [0.79, 1.74]	1.04 [0.50, 2.16]	0.89 [0.47, 1.67]
CVD	1.12 [0.76, 1.66]	1.00 [0.56, 1.80]	0.89 [0.56, 1.41]
Fatal CVD	1.47 [0.67, 3.26]	0.68 [0.12, 3.84]	0.46 [0.10, 2.19]
Stroke	1.32 [0.50, 3.51]	0.64 [0.12, 3.34]	0.49 [0.12, 2.06]
Heart Failure	1.03 [0.65, 1.64]	0.65 [0.28, 1.52]	0.63 [0.30, 1.31]
Kidney Failure	1.20 [0.76, 1.88]	0.98 [0.47, 2.03]	0.82 [0.45, 1.48]
Hypoglycaemia	1.46 [0.45, 4.72]	1.33 [0.22, 8.20]	0.91 [0.22, 3.82]
Hyperglycaemia	3.32 [0.46, 24.19]	N/A	N/A

Weighted Cox proportional hazards analysis. Estimated hazard ratios with 95% confidence intervals. * *p*-value <0.05

## Discussion

This observational study of 17,620 patients with type 2 diabetes monitored for up to 6.4 years provides information about the long-term effectiveness and safety of RAIs. The impact on weight and glycemic control was very similar between the three RAI groups. Overall, there were no differences in the risks of mortality or hospitalization due to CVD, CHF or renal failure using weighted Cox proportional hazards models, particularly among patients with impaired renal function. There seems, however, to be a significantly lower relative mortality risk among patients on insulin glulisine than on insulin aspart, as well as lower risk of stroke among users of glulisine in the total cohort and age 65 or older. Similarly, the risk of severe hyperglycemia was higher among lispro than aspart users, as well as of severe hypoglycemia than among both aspart and glulisine users in the older age group. The results are generally consistent with our previous report of type 1 diabetes [7] but for a larger group of patients with type 2 diabetes, who are at higher cardiovascular risk.

The results suggest a beneficial effect of insulin glulisine on overall mortality and stroke, but there were no differences in terms of fatal CHD or CVD between any of the groups. It is highly unlikely that other mechanisms would cause such an effect, such as, e.g., differences in pharmacokinetic or pharmacodynamics profiles, vascular effects or mitogenic activity [2, 7]. As in most observational studies, residual confounding is likely to be a contributing factor. Nevertheless, the major strengths of this study are its nationwide scope and statistical methods, which take all available clinical characteristics and treatment options into consideration.

The interpretation of the results is complicated by the effects of other risk factors and treatments, such as the use of antihypertensive, antihyperlipidemic or glucose-lowering agents, all of which can change with time. We did, however, take all baseline characteristics and risk factor treatments into account in the weighted Cox regression analyses. Given that the patients are encouraged to frequently adjust the dosage of the various insulin injections on their own during the follow-up period, we have not analyzed the possible role of dose size. Moreover, almost all patients used basal insulin. We did, however, only include patients not previously treated with RAI in order to rule out the effect of previous use of various insulin preparations that had been marketed on different dates.

Current glucose-lowering treatments, such as SGLT-2 inhibitors, DPP-4 inhibitors and GLP-1 receptor analogs, have been examined with regard to long-term safety in major randomized clinical outcome trials [15–17]. The ORIGIN trial showed that insulin glargine is non-inferior to other pharmacological treatments with respect to cardiovascular outcomes, cancer and death in individuals with type 2 diabetes and high cardiovascular risk [18]. But data are not yet available from any additional prospective studies concerning the long-term safety of other types of insulin. Post-marketing surveillance and observational cohort studies are needed to monitor the effects of older treatments in clinical practice. Such studies can provide data quality comparable to that of randomized control trials, which are usually limited by poor external validity [19].

## Conclusion

Our conclusion is that there do not appear to have been any major clinically important differences in effects on hypoglycemia, hyperglycemia, weight or long-term safety between the three available RAIs among insulin-naive individuals with type 2 diabetes who were monitored for up to 6.4 years in clinical practice.

## Abbreviations

BMI: Body mass index
CHD: Coronary heart disease
CHF: Congestive heart failure
CVD: Cardiovascular disease
eGFR: Estimated glomerular filtration rate
HbA1c: Glycosylated hemoglobin
PDR: Prescribed drug register
RAI: Rapid-acting insulin analog
